# New insight on the assessment of potassium silicate on pharmaceutical, morphological, and elemental status of *Ficus carica*: in vitro

**DOI:** 10.1186/s13104-026-07671-3

**Published:** 2026-03-05

**Authors:** Nesreen Houssien Abou-Baker, Doha H. Aboubaker, Nabil S. Mustafa, Rania A. Taha

**Affiliations:** 1 Soils and Water Use Department, Agriculture and Biological Research Institute, National Research Centre, Dokki, Giza Egypt; 2 Medicinal and Aromatic Plants Department, Pharmaceutical and Drug Industries Institute, National Research Centre, Dokki, Giza Egypt; 3 Biotechnology and Micropropagation Lab, Pomology Department, Agriculture and Biological Research Institute, National Research Centre (NRC), Dokki, Giza Egypt; 4 Tissue Culture Technique Lab, Central Laboratories Network, NRC, Dokki, Giza Egypt

**Keywords:** Antioxidant activity, Element contents, Ficus carica, Silicon, Fig shoot extracts, Free radical scavenging activities

## Abstract

**Background and objective:**

Recently, silicon (Si) has been demonstrated to be an important element in agriculture. Its significance in plant growth has prompted numerous studies. However, the knowledge about its role in plant tissue cultures is still limited. In an attempt to understand the role and effect of silicon on plant growth, morphologically and physiologically, in vitro, regenerated fig (*Ficus carica* var. Sultany) was used to study this purpose.

**Materials and methods:**

Silicon as potassium silicate (K_2_O.4SiO_2_; PS) was added to the MS medium at 0 (PS0), 0.1 (PS1), 1.0 (PS2), and 10.0 (PS3) mg L^− 1^. After four subcultures, morphological measurements and contents of element compounds, including N, P, K, Na, Ca, Fe, Mn, Zn, Cu, Ni, Cr, Cd, Pb, and As, were determined in treated shoots, high-performance liquid chromatography (HPLC) was used to identify and quantify phenolic compounds, total phenolic, and flavonoid contents, while free radical scavenging activities were determined using 2,2-diphenyl-1-picrylhydrazyl (DPPH) and 2,2′-azino-bis(3-ethylbenzothiazoline-6-sulfonic acid) (ABTS) assays.

**Results:**

Results showed that adding silicate at 1.0 mg L^− 1^ enhanced shoot number, leaf number, chlorophyll, growth vigour, leaf area, fresh and dry weight, and significantly reduced vitrification. Nineteen compounds were identified, with fig shoot extracts showing significant variation in the contents of phenolic acids and flavonoids.

**Conclusion:**

The use of potassium silicate enhanced fig growth, elemental content, and pharmacological components; however, a high rate (10.0 mg L^− 1^) diminished most parameters, with the exception of Na and Cr. Further studies using low rates of traditional and nanoparticles of silicate are recommended.

## Introduction

Tissue culture is a widely used environmentally friendly technique for mass propagation of economically important plant species [1–4]. The in vitro response of plant species mainly requires a culture medium enriched with macro and micro-nutrients, which have previously been extensively reported. For example, Murashige and Skoog (MS) medium [5] is a common nutrient medium used in all micropropagated plants. However, micropropagated plants on MS medium show several physiological problems, such as browning, hyperhydricity (vitrification), and shoot-tip necrosis [6]. In vitro, regenerated plantlets often also exhibit low survival rates during the acclimatization process due to transplantation shock caused by the effects of harsh environmental conditions on plantlets with weak survival properties. Therefore, improving plantlet acclimatization properties for increased transplantation success is warranted [7].

Silicon has increasingly become a crucial element for ensuring improved and sustainable crop production under the current impacts of global climate change. Although Si is not an essential element for higher plants [8], it is one of the most beneficial elements for plants, particularly during stress conditions [9]. Consequently, many studies have suggested that silicon should be added to the list of essential elements [10–12]. Silicon promotes the growth of various higher plants [13], plant element uptake, as well as alleviation of biotic and abiotic stresses [9, 14]. Silicon enhances plant-salt tolerance by raising water uptake and antioxidant enzyme activity or reducing lipid peroxidation [15, 16]. Several studies supported the use of potassium silicate (PS) as an effective source of Si and K and its benefits in promoting plant growth and nutrient uptake [17, 18]. A recent in vitro study reported that the addition of 200 mg/L PS significantly improved kalmegh (*Andrographis paniculata*) growth, pod count, and its health [19]. The application of PS, which does not contain volatile organic compounds nor release ecologically harmful byproducts, was demonstrated to mitigate the negative effects of environmental stresses associated with the yield of numerous plants [20]. Treatment with silicon solutions (potassium silicate) rather than sulfate solutions significantly increased all measured parameters of the bean plant, producing the highest basic branch, pod number, biological yield, stover yield, and plant height [10, 11]. All moringa (*Moringa oleifera* Lam) growth characters increased with the application of a mixture of silica and salicylic acid [21]. Treatment of biofuel plants with silicate could be vital for mitigating the harmful effects of irrigation with seawater [12]. Si was also able to improve plant growth conditions by increasing the content of total phenols (TP) and flavonoids (TF), especially in the roots, and reducing the content of antioxidant enzymes activity in tissue culture plants [2, 22]. In addition, a previous study confirmed that Si is one of the most essential elements for plant growth under stress conditions [11].

Knowledge about the role of Si in plant tissue culture studies remains limited as its application in commercial tissue culture labs. Many problems in the plant tissue culture process may be overcome with Si application. Furthermore, the MS culture medium may need some modifications to produce healthy plantlets and may include the addition of Si. Thus, the overall aim of this study was to evaluate the possible effects of potassium silicate on shoot induction, biomass formation, and elemental status (N, P, K, Na, Ca, Fe, Mn, Zn, Cu, Ni, Cr, Cd, Pb, and As), as well as to determine TP and TF contents in fig regenerated plantlets. In addition, free radical scavenging activities (DPPH and ABTS) were also determined in the shoot cultures.

## Materials and methods

### Study area

This study was conducted at the Tissue Culture Technique Lab., Central Laboratories Network, Soils and Water Use Department, Agriculture and Biological Research Institute, Medicinal and Aromatic Plants Department, Pharmaceutical and Drug Industries Institute, National Research Centre, Dokki, Giza, Egypt. It took two seasons for culture and biochemical analysis. The study was conducted in 2022–2023.

### Material preparation

 In vitro, shoot tips of the ‘Sultany’ fig cultivar derived from establishment stages were individually cultured in MS medium supplemented with 0.5 mgL^− 1^6-benzylaminopurine (BAP), 30 g L^− 1^ sucrose, and 6 g L^− 1^ Difco Bacto agar [23, 24]. The pH of the medium was adjusted to 5.7, then autoclaved at 121 °C and 15 Ib/in^2^ for 20 min.

### Experimental conditions

Silicon as potassium silicate (K_2_O.4SiO_2_; PS) was added to growth MS medium at concentrations of 0.0 (PS0), 0.1 (PS1), 1.0 (PS2), and 10.0 (PS3) mg L^− 1^. PS powder was assessed using the LEO-1530 scanning electron microscope (SEM model LEO Co., Germany) and energy diffraction X-ray (EDX) spectroscopy (Oxford Co., England). The cultured explants were incubated at an average temperature of 25 ± 2 °C under 16 h of artificial light intensity of about 1500 lx generated by white fluorescent lamps, followed by eight hours of darkness. Subculturing was performed four times at four-week intervals.

### Morphological parameters

After the 4th subculture from the start, morphological properties, such as average shoot number, shoot length (cm), leaf number, chlorophyll, growth vigour, leaf area, vitrification (1 = no vitrification, 2 = slight, 3 = medium, 4 = high and 5 = very high vitrification), fresh weight (FW), and dry weight (DW) g/ cluster were measured and recorded.

### Chemical contents

Shoot tissue samples were digested with sulphuric and perchloric acids using Kjeldahl Digestion Unit. The concentrations of N, P, K, Na, Ca, Fe, Mn, Zn, Cu, Ni, Cr, Cd, Pb, and As were determined as previously described [25]. Nitrogen, P, and K were detected using the Microkjeldahl apparatus (C. Gerhardt GmbH & Co. KG, Germany) ascorbic acid method and photometrically with the PFP7-JENWAY-flame-emission photometric apparatus (UK), respectively, while the remaining elements were determined using the plasma device (Agilent 5100 Synchronous Vertical Dual View (SVDV) ICP-OES and the Agilent Vapor Generation Accessory VGA 77, Australia) [25].

### Measurement of biochemical contents

Extraction and identification of phenolic and flavonoid compounds, as well as quantification of total phenols (TP), total flavonoids (TF), and antioxidant activity of fig clusters were performed as follows:

### Extraction of phenolic compounds

About 500 mg of the shoot sample was used for phytochemical analysis. The crushed powder was mixed with 10 mL of 70% methanol, and the solution was placed on a shaker at 24 rpm for 24 h at 25–30 °C, followed by sonication and vortexing for 30 min, then a final sonication for 15 min [26]. The mixture was then centrifuged at 6500 rpm for 10 min. Subsequently, the supernatant was collected, then the syringe filtered, and transferred to new Eppendorf tubes afterwards, kept at 4 °C for the phytochemical analysis.

### Identification of phenolic and flavonoid compounds using HPLC

Analysis with HPLC was conducted (1260 series HPLC instrument, Agilent Technologies, USA). The compounds were separated on an Eclipse C18 column with 4.6 mm x 250 mm i.d., 5 μm. Water (A) and trifluoroacetic acid at 0.05% in acetonitrile (B) were the mobile phase (a flow rate of 1 mLmin^‒ 1^). The mobile phase was programmed consecutively in a linear gradient as follows: 0 min (82% A); 0–5 min (80% A); 5–8 min (60% A); 8–12 min (60% A); 12–15 min (85% A), and 15–16 min (82% A). The multi-wavelength detector was monitored at 280 nm. Each sample solution was injected at 10 µL. The column temperature was remained at 35 °C.

### Determination of TP and TF contents as well as antioxidant activity of fig clusters

The fig cluster content of TP was determined using the Folin–Ciocalteu reagent with gallic acid (GAE) as a standard [27]. Meanwhile, TF content was evaluated using the aluminium chloride colourimetric method [28]. Catechin (CE) was utilized to create the calibration curve. In addition, free radical scavenging ability (DPPH and ABTS) was measured using the technique described by Hwang and Thi [29].

### Data analysis

The treatments were repeated three times each replicate contains three jars. The experiment was arranged in one Way Completely Randomized design (1WCR). Three uniform Jars were selected from each treatment for estimation (*n* = 3). The measured values were statistically analyzed by ANOVA [30] using the COSTAT program version 6.3.0.3. Duncan’s Multiple Range Test was used to compare the differences of means. Different alphabetical letters (for each parameter) represent significantly different means at a 5% (*p* < 0.05) level for morphological parameters and at 1% (*p* < 0.01) for pharmaceutical and elemental analysis.

## Results

### Evaluation of potassium silicate (PS) morphology using SEM and EDX assays

**Fig. 1:**
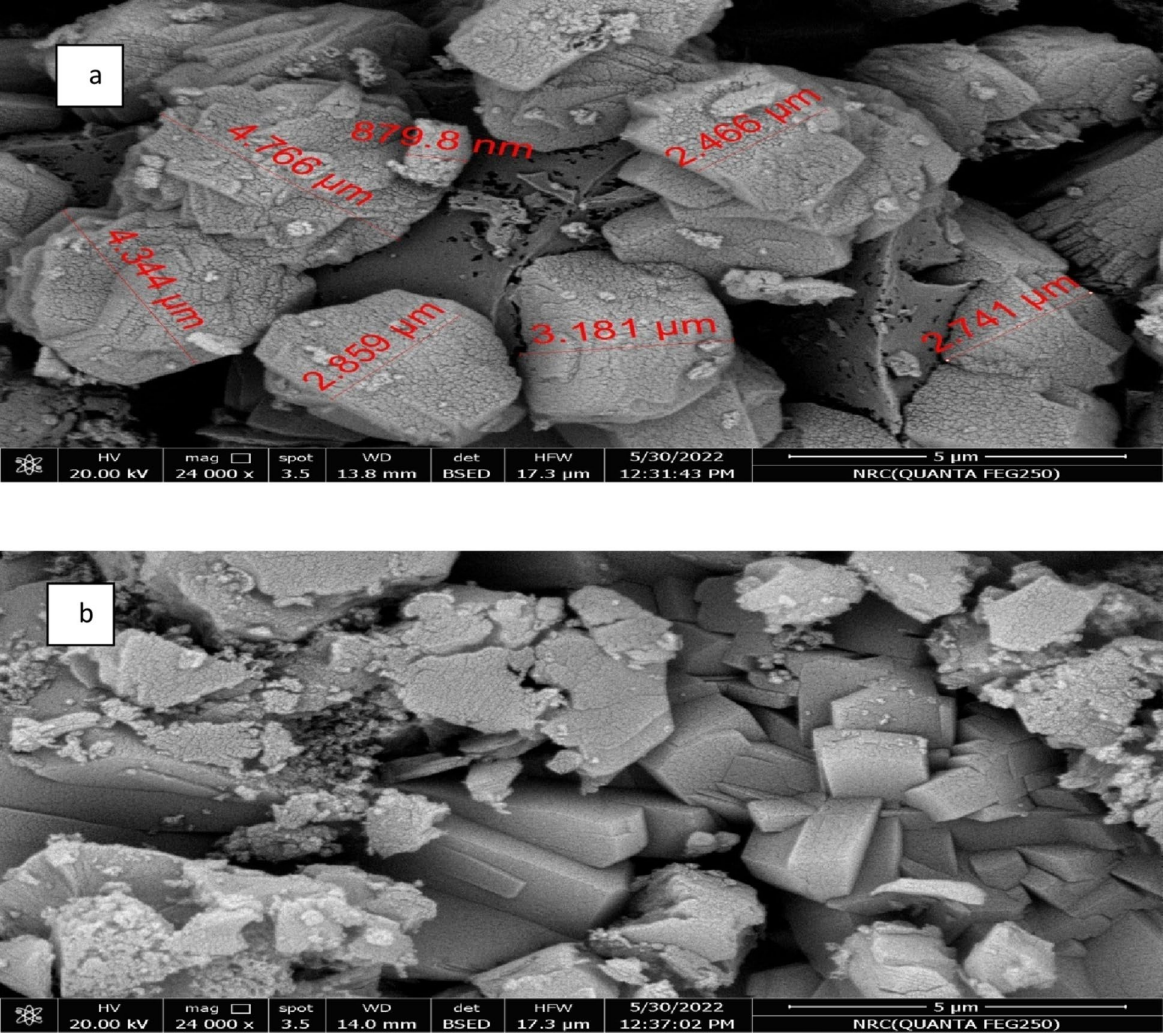
SEM micrographs of PS showing irregular rocky granules (**a**) and long rectangular-shaped features (**b**)

SEM was used to analyze the surface morphologies of PS, and the observed differences under 5 μm magnification are shown in Fig. 1. The SEM micrographs can detect sample particle size, surface bumps, crystal morphology, and surface roughness. The shape of PS crystals had irregular rocky granules (Fig. 1a) with some long rectangular-shaped features (Fig. 1b). The surfaces of rocky granules contained crowded cracks, while the surfaces of the rectangular-shaped features were smooth. The morphological differences were visible when the surface was amplified at high resolution (Fig. 1a and b). The inter-junction zone between the granules had numerous gaps and cavities, while particle sizes ranged between 879.8 nm and 4.766 μm.

**Fig. 2:**
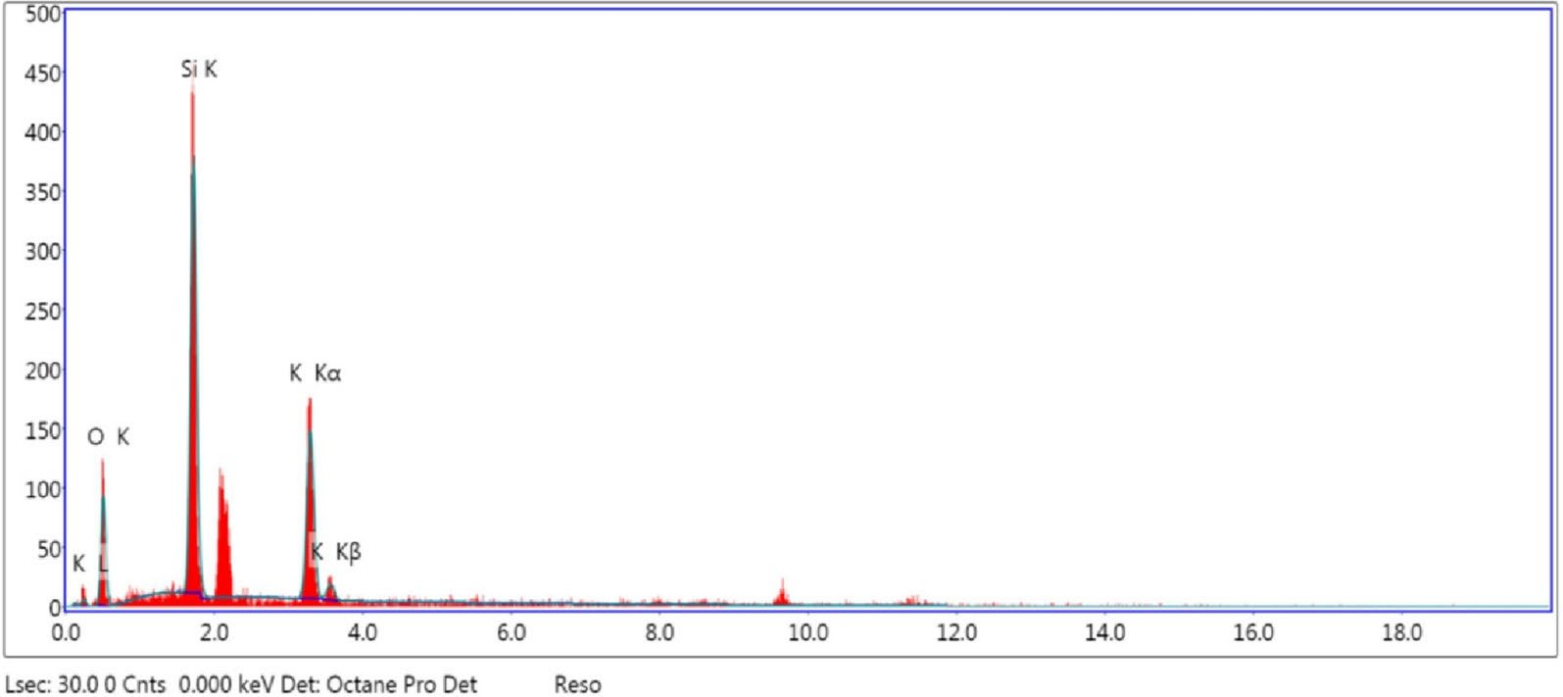
EDX spectra of PS showing structures of O, Si, and K atoms

Elemental analysis to evaluate the chemical structure of PS was performed using EDX spectroscopy, and the structures of O, Si, and K atoms were shown in Fig. 2. The percentage weight (%) values of these primary components (oxygen, silicon, and potassium) were 44.66, 31.89, and 23.44%, while the atomic percentage (%) values were 61.67, 25.09, and 13.25%, respectively. The absence of other elements demonstrated that PS had no impurities. An increased percentage of oxygen atoms may be attributed to the presence of potassium and silicon in oxide form (K_2_O.4SiO_2_).

### Morphological study of in vitro fig clusters treated with PS

**Fig. 3:**
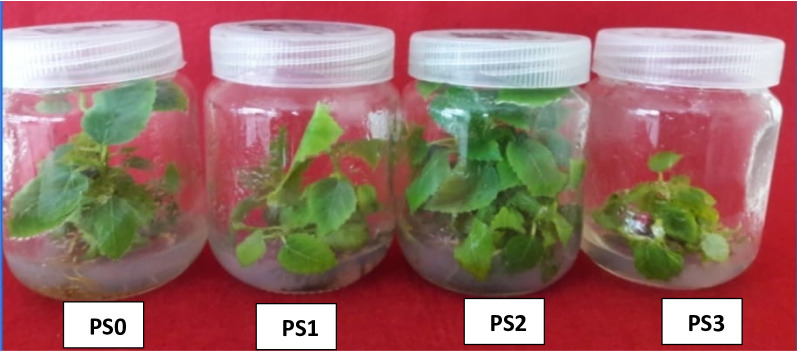
Effects of potassium silicate (PS) concentration on fig clusters morphology. Fig clusters induced in MS medium supplied with different concentrations of PS (PS0: 0.0, PS1: 0.1, PS2: 1.0, and PS3: 10.0 mg L^− 1^)

Potassium silicate (PS) application enhanced most morphological properties of in vitro fig clusters (Table 1). Notably, all silicate concentrations added to the culture medium significantly increased the average shoot number compared to the control (PS0), while PS2 treatment showed significant values for the highest shoot and leaf number, chlorophyll, growth vigour, and leaf area. In contrast, the highest silicate concentration (PS3) showed the lowest leaf number, chlorophyll, growth vigour, and leaf area values (Fig. 3).

**Table 1:** Effects of potassium silicate concentration on in vitro culture of fig clusters

PS (mg L^− 1^)	Shoot no.		Shoot length (cm)	Leaf no.		Chlorophyll (score)		Growth vigour (score)		Leaf area (score)		Vitrification (score)	
PS0	5.75^c^		3.35^a^	6.13^b^		2.75^c^		3.00^c^		3.00^bc^		5.00^a^	
PS1	7.00^b^		3.25^a^	6.32^b^		3.25^b^		3.50^b^		3.25^b^		3.00^c^	
PS2	11.75^a^		3.17^a^	7.88^a^		3.75^a^		5.00^a^		5.00^a^		2.00^d^	
PS3	6.75^b^		3.00^a^	5.49^c^		2.50^d^		2.00^d^		2.50^c^		3.50^b^	

Different alphabetical letters (for each column) represent significantly different means at 5% level

### Effect of PS treatment on the dry and fresh weight of fig clusters

**Fig. 4:**
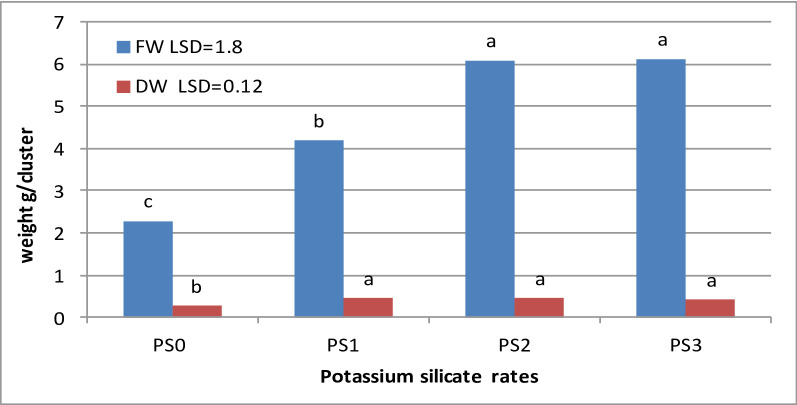
FW and DW values of fig clusters under different PS concentrations (PS0: 0.0, PS1: 0.1, PS2: 1.0, and PS3: 10.0 mg L^− 1^). Different alphabetical letters (for each parameter) represent significantly different means at 1% level

The addition of PS to the multiplication medium increased the FW and DW of fig clusters, with PS2 and PS3 indicating the highest FW followed by PS1, while the lowest value was observed in the untreated PS0 (the control) (Fig. 4). No significant difference was observed in FW values of PS2 and PS3 concentrations. Although the application of potassium silicate significantly increased the DW relative to the control, no significant differences were detected in its values between treatments PS1, PS2, and PS3.

### Profiles of macro-elements in fig clusters under PS treatments

Potassium silicate had similar effects on N and P concentrations, and their profiles under treatment followed the order of PS1 > PS0 > PS2 > PS3 (Table 2). The highest and the lowest potassium concentrations were detected in clusters of PS1 and PS0, respectively, while no significant difference in their concentration was observed between PS2 and PS3. In contrast, sodium percentage (%) and content (mg/cluster) followed the order of PS3 > PS2 = PS1 > PS0, while Ca% and content followed the order of PS1 > PS2 > PS3 > PS0, which was similar to those observed for the contents of N, P, and K. Overall, the control had the lowest values of macro-elements, while PS application at PS1 produced the highest values, with few differences being observed between PS2 and PS3 treatments (Table 2).

Iron, Mn, and Zn concentrations had similar trends with those of N, P, K, and Ca, as described above (Tables 3 and 4). Copper concentration decreased with the addition of PS, and its lowest value was detected in the PS3 treatment. The lowest Ni concentration was observed in the PS2 treatment, while content in other treatments was similar (0.011 ppm). Both PS1 and PS2 treatments increased Cr concentration compared with those of control and PS3 treatments. Cd, Pb, and As elements were not detected (ND) (Table 3), and a discrepancy was observed in the trends of Cu, Ni, and Cr contents.

**Table 2:** Percentage concentrations of macro-elements and content Mg clusters^- 1^

PS (mg L^− 1^)	Concentration (%)					Content (mg clusters^-1^)				
	*N*	*P*	K	Na	Ca	*N*	*P*	K	Na	Ca
PS0	2.839^b^	0.117^b^	2.026^c^	0.089^b^	0.036^c^	7.666^c^	0.316^b^	5.472^c^	0.240^b^	0.091^c^
PS1	3.620^a^	0.291^a^	3.600^a^	0.124^ab^	0.219^a^	16.296^a^	1.309^a^	16.200^a^	0.558^a^	0.979^a^
PS2	2.766^c^	0.107^c^	2.926^b^	0.124^ab^	0.142^b^	12.448^b^	0.480^b^	13.169^ab^	0.567^a^	0.646^b^
PS3	2.182^d^	0.088^d^	2.963^b^	0.160^a^	0.134^b^	9.164^bc^	0.369^b^	12.431^b^	0.671^a^	0.561^b^

Different alphabetical letters (for each column) represent significantly different means at 1% level

**Table 3:** Micronutrients and heavy metal concentrations (ppm) in shoot samples according to PS treatments

PS (mgL^− 1^)	Fe	Mn	Zn	Cu	Ni	Cr	Cd	Pb	As
PS0	1.205^d^	0.931^c^	0.771^c^	0.051^a^	0.011^a^	0.001^c^	ND	ND	ND
PS1	2.305^a^	1.681^a^	1.208^a^	0.021^c^	0.011^a^	0.007^b^	ND	ND	ND
PS2	2.105^b^	1.381^b^	0.851^b^	0.041^b^	0.001^b^	0.011^a^	ND	ND	ND
PS3	1.405^c^	1.381^b^	0.608^d^	0.011^d^	0.011^a^	0.001^c^	ND	ND	ND

Different alphabetical letters (for each column) represent significantly different means at 1% level. ND: No detection

**Table 4:** Some microelements content µg/cluster according to PS treatments

PS (mg L^− 1^)	Fe	Mn	Zn	Cu	Ni	Cr
PS0	0.325^c^	0.251^c^	0.208^c^	0.014^b^	0.003^b^	0.0003^c^
PS1	1.037^a^	0.756^a^	0.543^a^	0.009^b^	0.005^a^	0.0032^b^
PS2	0.947^a^	0.621^ab^	0.383^b^	0.018^a^	0.001^c^	0.0050^a^
PS3	0.590^b^	0.580^b^	0.255^c^	0.005^c^	0.005^a^	0.0004^c^

Different alphabetical letters (for each column) represent significantly different means at 1% level

### Pharmacological analysis

The effects of silicate supplementation in cell culture medium on the accumulation of plant secondary metabolites of fig clusters, including TP and TF contents, free radical scavenging activities (ABTS and DPPH), as well as phenolic acid and flavonoid contents were evaluated using HPLC (Table 5).

### Secondary metabolite production

**Fig. 5:**
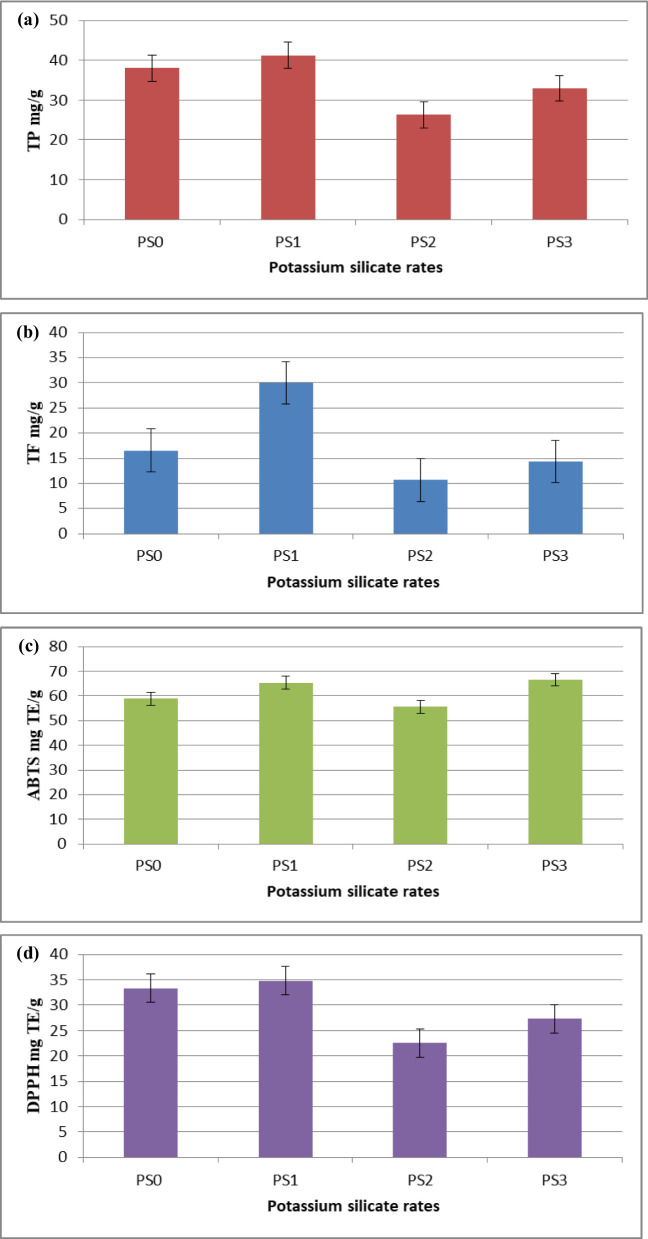
TP (**a**) and TF (**b**) contents and antioxidant activities (**c** and **d**) of fig extracts according to PS treatments (PS0: 0.0, PS1: 0.1, PS2: 1.0, and PS3: 10.0 mg L^− 1^)

TP (Fig. 5a), TF (Fig. 5b), and free radical scavenging activities (ABTS and DPPH; Fig. 5c and d, respectively) were studied in fig clusters treated with different concentrations of silicate. As a result, the lowest PS concentration was shown to promote higher levels of secondary metabolite production. A significant increase in the TP and TF contents increased the antioxidant activity.

### Identification and quantification of phenolic compounds in the extracts of fig clusters using HPLC

To identify and quantify phenolic compounds from fig extracts, HPLC peaks obtained were compared with the UV absorption spectra. Results showed significant variation in the contents of phenolic acids and flavonoids, with chlorogenic acid as the most important phenolic compound in the fig extract followed by Rutin (Table 5).

Three phenolic compounds, including Gentisic, Quercetin, and Kaempferol were undetected in fig extracts, which suggested that they were not affected by PS application, while the contents of p-hydroxybenzoic, Caffeic, and Vanillic did not increase with PS treatment. In addition, nine phenolic compounds, including Gallic, Protocatechuic, Cateachin, Chlorogenic, Syringic, Ferulic, Sinapic, p-coumaric, and Rutin were detected at low concentrations, which increased with PS concentration. Only four compounds, Rosmarinic, Cinnamic, Apigenin-7-glucoside, and Chrysin were decreased with increased PS concentration.

**Table 5:** Composition of phenolic compound in the extract of fig clusters according to PS treatments

Compounds	RT (min)	Phenolic content (ugg^-1^)			
		PS0	PS1	PS2	PS3
Gallic	3.9	215.18	353.97	291.31	357.82
Protocatechuic	7.65	64.34	81.92	108.95	77.73
p-hydroxybenzoic	11.88	ND	ND	626.38	664.58
Gentisic	11.8	ND	ND	ND	ND
Catechin	14.74	83.78	842.29	268.19	825.46
Chlorogenic	15.95	36.67	77.11	2726.99	4633.53
Caffeic	16.79	ND	1828.16	678.26	727.54
Syringic	18.67	93.97	258.92	187.84	51.54
Vanillic	20.55	ND	78.30	ND	53.65
Ferulic	28.27	28.27	61.82	44.21	68.87
Sinapic	30.156	324.97	1317.12	1201.58	1460.53
p-coumaric	34.4	119.83	62.52	50.62	328.42
Rutin	33.98	208.35	1118.05	1060.51	1330.16
Apigenin-7-glucoside	37.93	148.88	132.73	103.97	43.37
Rosmarinic	38.8	33.37	82.97	ND	ND
Cinnamic	46.12	20.61	47.83	ND	ND
quercetin	48.02	ND	ND	ND	ND
Kaempferol	55.06	ND	ND	ND	ND
Chrysin	59.03	216.06	44.43	61.04	12.80

*RT* Retention time,* ND* No detection

## Discussion

### Morphological study of in vitro fig clusters treated with PS

All PS treatments obviously reduced the hyperhydricity (vitrification) of fig shoots under in vitro conditions. The lowest vitrification value was observed with PS2 treatment. Interestingly, Si application was not only previously shown to cause maximum total leaf chlorophyll accumulation and number of shoots insapota cv. Kalipatti, but its role as a crucial element with numerous benefits to plants was also demonstrated. For example, Si was associated with plant growth and development, improved plant cell walls, and increased plant thickness, and it was also found to impart cell wall rigidity and decrease cell plasticity to enable tissue expansion, impart a dark green colour to the leaves, and enhance photosynthetic activity by increasing chlorophyll content [31]. In another study using potassium silicate improve the vegetative growth of chicory (*Cichorium intybus L.*) plant in vitro [17].

### Effect of PS treatment on weight and elemental analysis of fig clusters

Potassium and silicon elements in the PS compound promote plant growth [13] and nutrient uptake [10]. Although most morphological traits reduced at the PS3 rate, the fresh and dry weights showed high values. This finding could be attributed to the plant’s tendency to produce callus rather than the number and length of branches or the number and area of leaves as a protection mechanism against excessive chemical concentrations in the media. Potassium silicate application is an effective way to reduce nutrient stress in plants, reflected in a higher fresh and dry weight of the produced fig clusters. This strategy is possible due to the physical effect caused by the deposition of the Si bilayer in the cell wall [32]. Sales et al. [33] showed that Si supply mitigates the nutrient deficiency effects, notably N and Ca, by lowering the electrolyte leakage, enhancing the membrane integrity, improving the chlorophyll, and boosting the photosynthetic process, consequently increasing the fresh and dry mass of the quinoa crop. Although PS rates improved the fig growth and element contents, its high rate decreased most parameters except Na and Cr. This may be due to occurring unfavorable chemical reactions by application of high rate of PS such as (1) Si polymerization, (2) increasing the pH value of the media, (3) imbalance of nutrient uptake [18, 19].

### Effect of PS treatment on pharmacological analysis of fig clusters

Several studies have previously reported the biological activities of polyphenols and flavonoids [34–41]. However, sustainable and standardized methods for maximizing the production of flavonoids and phenols with higher antioxidant capacities are desirable yet still lacking.

In recent years, there has been a lot of interest in the use of PS to increase the phenolic content of plant cell cultures. Because of their well-known antioxidant qualities and function in plant defense systems, phenolic compounds are essential for human nutrition as well as plant health. Application of silicate has been shown to have a beneficial effect on the synthesis of these bioactive chemicals in a variety of plant species.

The highest amounts of TP and TF were observed in fig extract treated with low-PS concentration. This result was consistent with previous observations on the beneficial effects of PS supplementation in plant cell culture [2, 22]. The use of potassium silicate has been found to enhance the production of phenolic compounds, especially those responsible for antioxidant activity. The increase in phenolic and flavonoid compounds in plants treated with silicate may be attributed to these substances indirectly improving the plants’ photosynthetic abilities, allowing for greater production of assimilates for synthesizing secondary metabolites.

Potassium silicate application was also previously shown to cause relevant biochemical effects on induced clusters, such as increased ascorbate and carotenoids as well as phenolic compounds. The accumulation of such antioxidant compounds can enhance plant physiological state, leading to reduced electrolyte leakage into the cells and hindered chlorophyll breakdown, which improves the overall plant photosynthetic efficiency [42].

According to a study by Mohammadi et al. [43], adding PS to chicory plants enhanced their growth and raised the amount of phenolic compounds in the tissues that were produced. This was ascribed to silicate’s function in augmenting the physiological reactions of the plant and its ability to alleviate stress. According to Kováčik et al. [44], PS may also increase the activity of phenylalanine ammonia-lyase, an enzyme that is essential for the synthesis of phenolic compounds and is part of the phenylpropanoid pathway.

Additionally, Feghhenabi et al. [45] assessed how foliar PS spray and seed priming affected antioxidant activities in wheat at different salinity levels, which may have improved growth. Notably, in wheat subjected to saline circumstances, foliar spray and seed priming successfully reduced reactive oxygen species, reducing oxidative damage, strengthening antioxidant defenses, and boosting nutritional content and enzyme activity.

Overall, the observed high contents of compounds in PS1 may be attributed to the role of K and Si in plants, which is consistent with previous studies. For example, the contents of chlorophyll, K, P, Mg, and Fe, as well as the fresh and dry weight of plants were significantly increased in plants treated with K than in controls. Potassium plays vital roles in plant physiological processes, such as reducing the frequency and severity of frost, drought, wilt disease, osmotic adjustment, improving water use efficiency, carbohydrate assimilation, increasing sugar and protein stomatal closure in newly synthesized cells, alleviating the toxic impacts of Na, and influencing the rate of nearly all biological systems in plants [46]. Similarly, studies have shown that Si application promotes the growth of most plant species under normal conditions or biotic and abiotic stressors [12, 16, 17]. This could be attributed to; (1) Si deposition in the form of amorphous silica that leads to reinforced cell walls, which limit transpiration (2) enhanced water use efficiency, nutrient accumulation, leaf erectness, element selectivity, nutrient transportation, K: Na ratio, photosynthetic activity, ultra-structure of chloroplasts, and chlorophyll content (3) alleviation of numerous stressors, nutrient imbalance, mutual shading, electrolytic leakage of the leaves, translocation of Na and Cl ions, and reduced metal toxicity, particularly of sodium [10–12]. Although potassium and silicon application can induce all the above-mentioned beneficial effects, high concentrations of potassium silicate reversed their benefits, while its concentration at 1.0 mg/L improved growth and resulted in greater grain yield and rice quality [47].

Considering the explained results, it was found that including PS in the culture medium is necessary for improving multiplication, growth vigour, leaf area, and chlorophyll and decreasing vitrification problems in fig in vitro cultures. It also increased secondary metabolite production, and hence, it is recommended in figs tissue culture. In chicory plants, the application of PS resulted in a reduction in malondialdehyde (MDA) while enhancing anthocyanin, flavonoid, total phenol, and essential nutrient content, particularly K. These compounds directly affect the production of protein, sugar, starch, and cell division and expansion [17].

Polyphenolic compounds play a significant role in enhancing the overall fitness of fruits [45]. The fruit quality parameters of Ficus callus improved following potassium silicate treatments. Our findings are aligned with those of Tesfay et al. [48], who found that avocado fruit was improved following silicon treatments. Therefore, Silicon applications could be used to increase phenolic compounds in fruit, thereby increasing fruit quality. Furthermore, polyphenolic compounds with high antioxidant activity have been shown to induce anticancer effects in various cell lines [49, 50]. Notably, this study marks the first instance of evaluating the effect of potassium silicate treatment on pharmaceutical profile of *Ficus carica* callus. Thus, this research serves as an essential resource for future studies examining the effects of potassium silicate on the pharmaceutical profiles of medicinal plants within pharmacological research.

## Conclusion

Although silicon (Si) has been demonstrated to be an important element in agriculture, its role in plant tissue culture processing is still not clear. According to the presented results, it seems that silicon is an important element in tissue culture medium. It improved growth, multiplication, rooting, and secondary metabolites. In this manner, it is recommended to extend studying silicon’s role in other economically important plants.

## Significance statement

Silicon application as potassium silicate enhanced the growth, morphology, nutrient status, and pharmacological component of the in vitro fig plants. Therefore, potassium silicate is an important salt to be added to various plant tissue culture media. There is a need to investigate silicon’s role in other plants and other targeted techniques such as somatic embryogenesis, micropropagation, and secondary metabolite production. To fully comprehend potassium silicate’s role in plants, studying the molecular mechanism of potassium silicate in cell developmental processes, plant secondary metabolism, and any potential future implications is recommended.

## Data Availability

The datasets used in this investigation are all included in this publication, and upon justifiable request, they are also available from the corresponding author.

## References

[1] Taha RA, Hassan MM, Ibrahim EA, Abou Baker NH, Shaaban EA. Carbon nanotubes impact on date palm in vitro cultures. Plant Cell Tiss Organ Cult. 2016;127:525–34. 10.1007/s11240-016-1058-6.

[2] Sahebi M, Hanafi MM, Azizi P. Application of silicon in plant tissue culture. Vitro Cell Dev Biol -Plant. 2016;52:226–32. 10.1007/s11627-016-9757-6.

[3] Gaafar AA, Taha RA, Abou-Baker NH, Shaaban EA, Salama ZA. Evaluation of regeneration, active ingredients and antioxidant activities in jojoba tissue cultures as affected by carbon nanotubes. Biosci Res. 2018;15:2383–2392. https://doi.org/https://www.isisn.org/BR15(3)2018/2383-2392-15(3)2018%20BR-272.pdf.

[4] Häkkinen ST, Nygren H, Nohynek L, Puupponen-Pimiä R, Heiniö RL, Maiorova N, Rischer H, Ritala A. Plant cell cultures as food—aspects of sustainability and safety. Plant Cell Rep. 2020;39:1655–68. 32892290 10.1007/s00299-020-02592-2 PMC7644541

[5] Murashige T, Skoog F. A revised medium for rapid growth and bioassays with tobacco tissue cultures. Physiol Plant. 1962;15:473–97. 10.1111/j.1399-3054.1962.tb08052.x.

[6] Bairu MW, Kane ME. Physiological and developmental problems encountered by in vitro cultured plants. Plant Growth Regul. 2011;63:101–3. 10.1007/s10725-011-9565-2.

[7] Hassan MM, Taha RA, Abd El-Aziz ME, Shaaban EA, Ibrahim EA. Impact of nano-zinc-oxide as an alternative source of zinc in date palm in vitro cultures. Plant Cell Tiss Organ Cult. 2022;150:73–84. 10.1007/s11240-022-02235-2.

[8] Teixeira GCM, Porras CVG, Ferreira PM, Rocha JR, Prado RdeM. Silicon and Nano-Silicon in water use efficiency. In: de Mello Prado R, Etesami H, Srivastava AK, editors. Silicon advances for sustainable agriculture and human Health. sustainable plant nutrition in a changing world. Cham: Springer; 2024. 10.1007/978-3-031-69876-7_13.

[9] Ma JF. Role of silicon in enhancing the resistance of plants to biotic and abiotic stresses. Soil Sci Plant Nutr. 2004;50:11–8. 10.1080/00380768.2004.10408447.

[10] Abou-Baker NH, Abd-Eladl M, Abbas MM. Use of silicate and different cultivation practices in alleviating salt stress effect on bean plants. Aust J Basic Appl Sci. 2011;5:769–81.

[11] Pavlovic J, Kostic L, Bosnic P, Kirkby EA, Nikolic M. Interactions of silicon with essential and beneficial elements in plants. Front Plant Sci. 2021;12:697592. 10.3389/fpls.2021.697592. 34249069 10.3389/fpls.2021.697592 PMC8261142

[12] Hussein MM, Al-Ashry SM, Haggag W, Abou-Baker NH. Micronutrients status of biofuel plant (Moringa) irrigated by diluted seawater as affected by silicate and Salicylic acid. Int J Eng Res. 2014;3:720–5. 10.17950/ijer/v3s12/1203.

[13] Zhu Z, Wei G, Li J, Qian Q, Yu J. Silicon alleviates salt stress and increases antioxidant enzymes activity in leaves of salt-stressed cucumber (*Cucumis sativus* L). Plant Sci. 2004;167:527–33. 10.1016/j.plantsci.2004.04.020.

[14] Currie HA, Perry CC. Silica in plants: biological, biochemical and chemical studies. Ann Bot. 2007;100:1383–9. 10.1093/aob/mcm247. 17921489 10.1093/aob/mcm247 PMC2759229

[15] Liang Y, Chen Q, Liu Q, Zhang W, Ding R. Exogenous silicon (Si) increases antioxidant enzyme activity and reduces lipid peroxidation in roots of salt-stressed barley (*Hordeum vulgare* L). J Plant Physiol. 2003;160:1157–64. 10.1078/0176-1617-01065. 14610884 10.1078/0176-1617-01065

[16] Zhu YX, Xu XB, Hu YH, Han WH, Yin JL, Li HL, Gong HJ. Silicon improves salt tolerance by increasing root water uptake in *Cucumis sativus* L. Plant Cell Rep. 2015;34:1629–46. 10.1007/s00299-015-1814-9. 26021845 10.1007/s00299-015-1814-9

[17] Mohammadi H, Abdollahi-Bastam S, Aghaee A, Ghorbanpour M. Foliar-applied silicate potassium modulates growth, phytochemical, and physiological traits in *Cichorium intybus* L. under salinity stress. BMC Plant Biol. 2024;24:288–97. 10.1186/s12870-024-05015-6. 38627611 10.1186/s12870-024-05015-6 PMC11020321

[18] Gonzalez-Porras CV, Teixeira GCM, Prado RDM, Ferreira PM, Palaretti LF, Oliveira KS. Silicon via fertigation with and without potassium application, improve physiological aspects of common beans cultivated under three water regimes in field. Sci Rep. 2024;14:2051. 10.1038/s41598-024-52503-8. 38267535 10.1038/s41598-024-52503-8 PMC10808205

[19] Ranjith KS, Jnanesha AC, Bharathkumar S, Sravya K, Lal RK. Unveiling the effect of foliar applied siliceous compounds on reducing seed shattering for the conservation of endangered Kalmegh (*Andrographis paniculata* (Burm. f.) wall. Ex Nees). Biocatal Agric Biotechnol. 2025;103700. 10.1016/j.bcab.2025.103700.

[20] Gomaa MA, Kandil EE, El-Dein AAMZ, Abou-Donia MEM, Ali HM, Abdelsalam NR. Increase maize productivity and water use efficiency through application of potassium silicate under water stress. Sci Rep. 2021;11:224. 10.1038/s41598-020-80656-9. 33420308 10.1038/s41598-020-80656-9 PMC7794573

[21] Hussein MM, Abou-Baker NH. Growth and mineral status of Moringa plants as affected by silicate and Salicylic acid under salt stress. Int J Plant Soil Sci. 2014;3:163–77.

[22] Sivanesan I, Park SW. The role of silicon in plant tissue culture. Front Plant Sci. 2014;5:571. 10.3389/fpls.2014.00571. 25374578 10.3389/fpls.2014.00571 PMC4204432

[23] Taha RA, Mustafa NS, Hassan SAM. Protocol for micropropagation of two ficus carica cultivars. World J Agricultural Sci. 2013;9(5):383–8. 10.5829/idosi.wjas.2013.9.5.1802.

[24] Mustafa NS, Hassan SAM, Taha RA. In vitro studies on growth and rooting of some Fig cultivars. Res J Pharm Biol Chem Sci. 2016;7:124–30. https://www.rjpbcs.com/pdf/2016_7(5)/[17].pdf.

[25] Jones JB. Laboratory guide for conducting soil tests and plant analysis.CRC press. Boca Raton London New York Washington, D.C.ISBN: 0-8493-0206-4. p382. 2001. file:///G:/… add%20to%20hard/LABORATORY%20GUIDE%20FOR%20CONDUCTING%20SOIL%20TESTS%20AND%20PLANT%20ANALYSIS.pdf.

[26] Khan MA, Abbasi BH, Ahmed N, Ali H. Effects of light regimes on in vitro seed germination and Silymarin content in Silybum Marianum. Ind Crops Prod. 2013;46:105–10. 10.1016/j.indcrop.2012.12.035.

[27] Singleton VL, Rossi JA. Colorimetry of total phenolics with phosphomolybdic-phosphotungstic acid reagents. Am J Enol Viticult. 1965;16:144–58.

[28] Willett WC. Balancing life-style and genomics research for disease prevention. Science. 2002;296:695–8. 10.1126/science.1071055. 11976443 10.1126/science.1071055

[29] Hwang ES, Thi ND. Effects of extraction and processing methods on antioxidant compound contents and radical scavenging activities of Laver (*Porphyratenera*). Prev Nutr Food Sci. 2014;19:40–8. 10.3746/pnf.2014.19.1.040. 24772408 10.3746/pnf.2014.19.1.040 PMC3999807

[30] Gomez KA, Gomez AA. Statistical procedures for agricultural research. Wiley; 1984.

[31] Lalithya KA, Hipparagi K, Thippeshappa GN. Effect of silicon and micronutrients on growth and yield attributes of Sapota cv. Kalipatti under hill zone. Crop Res. 2013;46:146–9.

[32] Barbosa FMP, Snyder GH, Fageria NK, Datnoff LE, Silva OD. Silicato de cálciocomofonte de silíciopara o Arroz de Sequeiro. Rev Bras Ciênc Solo. 2001;25:325–30. 10.1590/S0100-06832001000200009.

[33] Sales AC, Campos CNS, de Souza Junior JP, da Silva DL, Oliveira KS, de Mello R, Prado LPR, Teodoro PE, Teodoro. Silicon mitigates nutritional stress in Quinoa (*Chenopodium Quinoa Willd*). Sci Rep. 2021;11:14665. 10.1038/s41598-021-94287-1. 34282251 10.1038/s41598-021-94287-1 PMC8289834

[34] El-Gengaihi S, Mossa ATH, Refaie AA, Aboubaker DH. Hepatoprotective efficacy of *Cichorium intybus* L. extract against carbon tetrachloride-induced liver damage in rats. J Diet Suppl. 2016;13:570–84. 10.3109/19390211.2016.1144230. 26913368 10.3109/19390211.2016.1144230

[35] El-Gengaihi SE, Hamed MA, Abou-Baker DH, Mossa AT. Flavonoids from sugar beet leaves as hepatoprotective agent. Int J Pharm Pharm Sci. 2016;8:281–6.

[36] Ibrahim EA, Abou Baker DH, El-Baz FK. Anti-inflammatory and antioxidant activities of rhubarb roots extract. Int J Pharm Sci Rev Res. 2016;39:93–9.

[37] Salam MA, Ibrahim BM, El-Batran SE, El-Gengaihi SE, Baker DH. Study of the possible antihypertensive and hypolipidemic effects of an herbal mixture on l-name-induced hypertensive rats. Asian J Pharm Clin Res. 2016;9:85–90. 10.22159/ajpcr.2016.v9i5.12175.

[38] Allam SF, Soudy BN, Hassan AS, Ramadan MM, Abou Baker DA. How do mentha plants induce resistance against *Tetranychusurticae* (Acari: *Tetranychidae*) in organic farming? J. Plant Prot Res. 2018;58:265–75. 10.24425/122943.

[39] Abou Baker DH. Plants against Helicobacter pylori to combat resistance: an ethnopharmacological review. Biotechnol Rep. 2020;26:e00470. 10.1016/j.btre.2020.e00470. 10.1016/j.btre.2020.e00470 PMC7248673 32477900

[40] Abou Baker DH. An ethnopharmacological review on the therapeutical properties of flavonoids and their mechanisms of actions: A comprehensive review based on up to date knowledge. Toxicol Rep. 2022;9:445–69. 10.1016/j.toxrep.2022.03.011. 35340621 10.1016/j.toxrep.2022.03.011 PMC8943219

[41] Abou Baker DH. Can natural products modulate cytokine storm in SARS-CoV2 patients? Biotechnol Rep. 2022;35:e00749. 10.1016/j.btre.2022.e00749. 10.1016/j.btre.2022.e00749 PMC9181898 35702395

[42] de Mendonça AO, Tavares LC, Brunes AP, Monzón DLR, Villela FA. Acúmulo de silício e compostosfenólicosna parte aérea de plantas de trigoapós a adubaçãosilicatada. Biosci J. 2013;29. ID: biblio-946886.

[43] Mohammadi H, Abdollahi-Bastam S, Aghaee A, Ghorbanpour M. Foliar-applied silicate potassium modulates growth, phytochemical, and physiological traits in cichorium intybus L. under salinity stress. BMC Plant Biol. 2024;24(1):288. 10.1186/s12870-024-05015-6. 38627611 10.1186/s12870-024-05015-6 PMC11020321

[44] Kováčik J, Klejdus B, Bačkor M, Repčák M. Phenylalanine ammonia-lyase activity and phenolic compounds accumulation in nitrogen-deficient matricaria Chamomilla leaf rosettes. Plant Sci. 2007;172(2):393–9. 10.1016/j.plantsci.2006.10.001.

[45] Feghhenabi F, Hadi H, Khodaverdiloo H, Van Genuchten MT, Pessarakli M. Improving wheat (Triticum aestivum L.) antioxidative defense mechanisms against salinity stress by exogenous application of potassium silicate. J Plant Nutr. 2022;45(19):2887–905. 10.1080/01904167.2022.2067776.

[46] Rachappanavar V, Gupta SK, Jayaprakash GK, Abbas M. Silicon mediated heavy metal stress amelioration in fruit crops. Heliyon. 2024. 10.1016/j.heliyon.2024.e37425. 39315184 10.1016/j.heliyon.2024.e37425 PMC11417240

[47] Wissa MT. Impact of potassium silicate compound as foliar application on the growth, yield and grains quality of GIZA 179 rice cultivar. J Plant Prod. 2017;8:1077–83. 10.21608/jpp.2017.41115.

[48] Tesfay SZ, Bertling I, Bower JP. Effects of postharvest potassium silicate application on phenolics and other anti-oxidant systems aligned to avocado fruit quality. Postharvest Biol Technol. 2011;60:92–9. 10.1016/j.postharvbio.2010.12.011.

[49] Ulusu F. Exploring the therapeutic potential of microwave-assisted biosynthesized silver nanoparticles using Erica manipuliflora Salisb.: A comprehensive study on anticancer and antibacterial potentials. Particuology. 2024;95:212–22. 10.1016/j.partic.2024.09.018.

[50] Ulusu F, Şahin A. Changes in cytotoxic capacity, phenolic profile, total phenols and flavonoids of *Nigella Damascena* L. seed extracts under different liquid fertilization. South Afr J Bot. 2022;150:500–10. 10.1016/j.sajb.2022.08.010.

